# Notes of a Case of Cerebro-Spinal Meningitis

**Published:** 1864-07

**Authors:** D. W. Young

**Affiliations:** Aurora, Ill.


ARTICLE XXVIII.

### NOTES OF A CASE OF CEREBRO-SPINAL MENINGITIS.

By D. W. YOUNG, M.D., of Aurora, Ill.

Read before the Aurora City Medical Association.

GENTLEMEN:—Since the last meeting of our Association, I have seen and treated a case of cerebro-spinal meningitis, and I now respectfully ask your indulgence while I read you my notes of the case. I shall be particularly minute, and relate all the minutia of symptoms and treatment; hoping thereby to incite to discussion and, if possible, elicit some new idea and facts concerning this terrible disorder and its treatment. I have no special theory or suggestions to advocate. I shall only communicate facts, and allow you to draw your own inference. I have read everything that I can find in my medical books and journals, and regret that I can find only a little—that is tangible and useful in managing this disease. My reading has forced me to the conclusion that, a greater portion of our medical authors and older practitioners are unacquainted with the disease. Therefore, I claim that it behooves us to seek, investigate, and record every fact that we can find bearing upon this subject. I assure you, gentlemen, you will need all your knowledge when you come to treat cerebro-spinal meningitis.

Saturday, March 26, 1864. I was called over into the North end of Will County, to see Thomas C., a Scotch boy, aged 15 years. Although he was only 15 years old, he measured 6 feet and 2 inches, and was well-proportioned, in a word, he was a giant on a small scale. I arrived at his place of residence about 2 o'clock P.M., and found his parents, brothers, and sisters in a very high state of excitement. They informed me that they had buried one son, next younger than this one, a few days before, who had died with what the doctors called spotted fever; and that this boy was attacked and acted just as the other had in the commencement of the disease—and they apprehended the same result. They also informed me that they had not told the patient that they had sent for the doctor, but would now tell him of my arrival, and then I should see him. The father retired, and after a few moments returned and conducted me into a large well-ventilated bedchamber, where I found the young Scotch giant in bed, with the following symptoms:—skin moderately hot and moist; pulse 68 per minute, slow and soft; eyes red and suffused; tongue dry and covered with a thick yellow coat; respiration a little hurried; no cough nor pain in the chest; bowels have not moved for two days; urine free and very high colored; some slight pain and stiffness in occipital and cervical regions. He complained of a peculiar soreness and sensitiveness extending all over him. It seemed to cause an uneasiness even to touch his hair. He said that he had not been well for several days; appetite had been poor. He had slight chills, with more or less shifting pain in the extremities. A general uneasiness and listlessness. On Friday afternoon, he had a distinct chill, followed with fever and a good deal of pain in the head and back of his neck. Was restless during the night, and had slept but little. A few hours before my arrival he had vomited freely, which relieved the pain in his head decidedly, and he was feeling much better. Said he thought it had been very foolish to send for a doctor to see him, as he was feeling so much better now; and he thought should get right along without any further trouble. After having examined the case thoroughly, as I thought, and reflected upon the symptoms, I informed the parents and friends that I could not discover anything alarming in the symptoms there present; and that I really hoped their fears were ungrounded. It was my first case. I was not fully acquainted with the deceptive intermission of the disease. They said, that the other boy had acted just so, as well as all the other cases that had occurred in that neighborhood, and that they had all died; therefore, I determined to be cautious and guarded in my prognosis of the case.

I ordered them to soak his feet thoroughly in hot mustard water, and also to apply a large mustard sinapism to the nape of his neck and down the spine. And ℞. Hydg. sub murias, grs. xx., Soda bicarb., grs. xij., Pulv. rhei, grs. v. Mix, and give immediately. And left him Quinia sulph., grs. xxx., Pulv. Doveri, grs. xx. Mix, and divide into five powders, and give one powder every three hours after the bowels have moved. Keep a cloth, wet in cold water, on his head, and give him warm drinks. If the bowels do not move in four hours, give him an ounce of castor oil. Thus I left him, with instructions that if anything occurred to let me know immediately. About eight o'clock in the evening, a messenger came, running his horse, saying that he was much worse, and that they desired my attendance as quick as possible. I started immediately; and arrived at their house a little after two o'clock. When I was fully two rods from the house, I heard him screaming; and when I entered his chamber I found things very different from what I left them, a few hours before. Now, he was sweating profusely; head thrown back upon his neck, and the muscles of his neck so rigid that it was impossible to move his head without moving or turning his body also; pulse variable, sometimes 80 to 90 per minute, then again so frequent that I could scarcely count them—these alternations occurred quite often; eyes very red and suffused; pupils changeable, but unaffected by light—sometimes they were dilated largely, and then within a few moments would contract down almost to a point; respiration hurried and sighing; bowels have not moved; he has voided his urine twice since my first visit; the pains in his head and neck seem to be decidedly paroxysmal, they come on suddenly and then he clasps his head with both hands and screams terribly. Soon after I left, in the afternoon, they substituted a large blister for the mustard to the nape of the neck. The blister has drawn quite thoroughly and is well filled. He is about semi-comatose, very restless, swallows with difficulty. Scarrified thoroughly along the spine, below the blister, and drew from four to five ounces of blood; then covered the scarrified surface with a new poultice covered with powdered morphia; enclosed the head in powdered ice; gave an enema of soap suds, castor oil, and turpentine. Also, one of the quinine powders immediately. Also, x gtts. of fluid extract of belladonna and xv gtts. of fluid extract of hyoscyamus every two hours. Fed him freely of Bourbon whiskey saturated with chlorate of potassa. I know that my directions were followed punctually to the letter, as I did not leave the bedside of my patient at all, but watched everything and every symptom closely all the time, determined to see if I could not detect something that might throw some new light upon this particular malady. 12 o'clock midnight.—All the symptoms about the same; bowels have not moved; repeated the enema; and continued the other treatment. 4 o'clock A.M.—No better; symptoms about the same; surface a little cooler, and a little blue denoting capillary congestion. Placed bottles filled with hot water about the patient and added capsicum to the prescription. Continued other medicines as before. Sunday morning, 8 o'clock.—No better; head is hotter and extremities colder. Repeated the cups to the spine and back of the head over the mastoid processes; repeat the enema. Retains the enemas, but the bowels do not act. He retains the medicines and stimulants well. No nausea. Still screams occasionally, and grasps his head with his hands frequently. Applied the cups to his temples, and drew blood freely. Continued the other treatment. 12 o'clock M.—Is no better; head symptoms are increasing; pupils are dilated, either from effusion or from the belladonna; he is more restless; bowels are rumbling freely; repeated the enema and continued the other treatment.

3 o'clock P.M.—Bowels have moved freely; he is more quiet and rational; skin warmer and moist; pupils still dilated; pulse 120 per minute; respiration sighing; do not think the muscles of the back of his neck are quite so rigid; complains of severe pain in the back of his head and neck; continue all the treatment.

Sunday evening, 6 o'clock.—Patient seems better; he has slept some, and is warmer and sweating freely; called for the vessel and voided his urine, which is very high colored, and has a decidedly ammoniacal smell; he says he feels better, and that his head does not ache much now; pupils still dilated; pulse irregular; respiration is slow but sighing; continue treatment.

Sunday night, 10 o'clock.—Patient not so well; head is hotter and surface colder; more restless and screams again; muscles of the neck not much rigid now; he throws himself about constantly; respiration irregular and sighing; does not answer questions as correctly; calls his friends, and says "come, let's go home." I repeated the cups; drew more blood, and continued all the other treatment energetically, but to no purpose. He grew worse; his head became hotter and thrown back again, and became more rigid than ever; had a few tetanic twitchings of the muscles, and died at 2 o'clock on Monday morning. I could not secure a *post mortem.* Thus, gentlemen, you have the particulars of the case and treatment. He died. Did I treat him right? Please give me your views of the case—and the disease.

